# The burden of work related musculoskeletal disorders among female beedi workers: a public health perspective

**DOI:** 10.3389/fpubh.2026.1879036

**Published:** 2026-07-30

**Authors:** Supriya Amanna, Yashodhar P. Bhandary, Ranajit Das, Rekha P. Shenoy, Apoorva Kotian

**Affiliations:** 1 Department of Public Health Dentistry, Yenepoya Dental College, Yenepoya (Deemed to be University), Mangaluru, Karnataka, India; 2 Yenepoya Research Centre, Yenepoya (Deemed to be University), Mangaluru, Karnataka, India

**Keywords:** beedi rollers, ergonomic risk factors, musculoskeletal disorders, occupational health, repetitive work, women workers

## Abstract

The beedi industry remains an important source of livelihood for millions of people in India, particularly women from economically disadvantaged backgrounds. Beedi rolling is a labor-intensive, home-based occupation that involves manually rolling tobacco flakes in tendu leaves. Although it offers flexibility and allows women to manage household responsibilities, the nature of the work exposes them to several occupational health risks. Among these, musculoskeletal disorders (MSDs) are one of the most common and persistent concerns, largely due to prolonged sitting, repetitive hand movements, and poorly designed work environments. This narrative review synthesizes existing evidence on musculoskeletal disorders among female beedi rollers in India. A literature search was conducted using PubMed, Scopus, and Google Scholar to identify relevant peer-reviewed studies. Studies focusing on female beedi workers and reporting musculoskeletal problems or related occupational risk factors were included. Data on study characteristics, participant profiles, prevalence of pain in different body regions, and ergonomic risk factors were extracted and analyzed. The findings indicate a consistently high burden of musculoskeletal problems, particularly affecting the lower back, neck, shoulders, wrists, and fingers. These conditions are closely associated with long working hours, sustained forward-bent postures, and repetitive movements over extended periods. The informal nature of this work further limits access to occupational health services and awareness of preventive measures. Addressing these issues requires simple ergonomic interventions, health education, and stronger policy support for informal sector workers.

## 1 Introduction

The beedi industry stands as one of the largest and most critical components of India’s informal economy, employing approximately 4.4 million workers across the country (1). The production of beedi, a labour-intensive cottage industry, entails rolling dried tendu leaves loaded with tobacco flakes by hand. This technique is still mostly unmechanised (2, 3). Millions of rural and impoverished households depend on the industry for their necessary income, but over the past three decades, it has shifted from factories to home-based production, putting it outside the purview of formal labour laws and safety inspections (1, 2).

The beedi industry is largely sustained by female workers, who make up nearly 90% of the total workforce (3). Women are commonly engaged in this occupation because the work requires patience and fine manual skills needed for rolling beedis. In addition, the home-based nature of the job often attracts women, as it allows them to combine income-earning activities with their household responsibilities. However, studies have shown that the time devoted to beedi rolling frequently surpasses the time spent on domestic work (4, 5).

Despite its economic importance, the largely informal structure of the beedi industry gives rise to significant occupational health concerns. Most workers carry out their tasks in environments that lack basic occupational safety measures, which exposes them to continuous tobacco dust and unfavorable working conditions such as poor ventilation and inadequate lighting (6, 7). Under these circumstances, musculoskeletal disorders have been reported as the most common health problem among workers, exceeding other conditions such as respiratory and skin-related ailments. Studies from similar informal occupational settings have reported pooled prevalence estimates of musculoskeletal disorders as high as 76% (7, 8). These disorders are largely attributed to the nature of the work, which requires workers to sit in a fixed posture for prolonged periods—often 8 to 10 h a day—while performing repetitive manual tasks (2). The piece-rate system of payment further intensifies this risk, as workers tend to increase their workload to meet production targets and maintain a basic level of income (9).

Given the high prevalence of these disorders and the continued lack of awareness among workers about the impact of their occupational activities on their health (2), the issue remains a significant concern. Although several regional studies have been conducted, the available evidence on musculoskeletal disorders among women beedi rollers is still fragmented. This highlights the need for a comprehensive synthesis of existing research. The present narrative review aims to bring together current evidence on musculoskeletal disorders among female beedi rollers in India, with a focus on the ergonomic and socioeconomic factors contributing to these conditions. These findings will support the development of effective occupational health interventions and policy initiatives.

## 2 Methodology

This review brings together current evidence on the prevalence of musculoskeletal disorders among female beedi rollers in India and highlights the ergonomic factors associated with these conditions, while examining the link between informal labor environments and physical health outcomes.

### 2.1 Literature search strategy

A comprehensive literature search was conducted across three major academic databases PubMed, Scopus, and Google Scholar (8, 10, 11) to identify relevant peer-reviewed studies published between 2005 and 2025, ensuring coverage of recent trends within India’s informal sector (8, 12). The search strategy incorporated a combination of keywords and Boolean operators. Population-related terms included “beedi workers,” “beedi rollers,” “female manual laborers,” and “informal sector workers in India” (11). Outcome-related terms comprised “musculoskeletal disorders,” “MSDs,” “prevalence,” “back pain,” “neck pain,” “wrist pain,” and “carpal tunnel syndrome.” Additionally, terms related to risk factors such as “ergonomic risk,” “occupational hazards,” “repetitive movements,” and “fixed posture” was used (10).

### 2.2 Inclusion and exclusion criteria

To ensure the relevance and quality of the synthesized data, specific inclusion and exclusion criteria were applied during the literature selection process. Studies were included if they focused primarily on female beedi workers or closely related manual cottage industries in India, such as handloom weaving and papad making, to provide a comparative ergonomic context (13, 14). Preference was given to studies reporting the prevalence of musculoskeletal disorders and those employing standardized assessment tools, particularly the Nordic Musculoskeletal Questionnaire (8). The review was further limited to peer-reviewed articles published in English (10, 13). Conversely, exclusion criteria were applied to studies focusing solely on male workers, those lacking specific musculoskeletal data, or works published prior to the 2005 cut off (8, 13). Additionally, studies addressing non-occupational sources of pain, such as pregnancy related conditions, were excluded to ensure the focus remained on work-related morbidity (6, 13).

### 2.3 Data extraction

Information was extracted from the selected studies into a standardized format for synthesis (8). The key data points extracted included study metadata such as author, year of publication, and geographical location (15); participant profiles encompassing sample size, age range, and years of occupational exposure; (2) morbidity prevalence featuring specific percentages of workers reporting pain in the lower back, neck, shoulders, and wrists (16); and ergonomic observations identifying risk factors such as duration of work, sitting posture, and repetitive hand motions (14).

## 3 Occupational profile of BEEDI rolling workers

The occupational profile of the beedi industry in India is fundamentally defined by its status as a highly gendered and decentralized segment of the informal economy. This sector is characterized by a nearly absolute dominance of female laborers, who constitute roughly 90% of the workforce (2). These women often originate from socioeconomically marginalized backgrounds, frequently belonging to “weak economic classes” or backward populations living in urban slums (4, 17). Many enter the trade due to limited formal education and a lack of specialized skills required for alternative employment, making beedi rolling a primary source of subsistence income (4, 18). The home-based nature of the work often referred to as a “putting-out” system is a defining characteristic; it allows women to navigate restrictive gender norms by balancing manual labor with domestic responsibilities, such as childcare and household chores, without leaving their homes (3–5). However, this “convenience” comes at a high structural cost, as the work operates within the informal sector’s “shadow,” where the absence of formal labor contracts and social security leaves workers vulnerable (1, 3). Furthermore, the industry relies on a piece-rate wage system that incentivizes long, unbroken hours of repetitive labor to meet high production targets, often leading to severe health complications that go unaddressed due to a widespread lack of awareness regarding government welfare initiatives and occupational safety standards (8, 18).

## 4 Prevalence of musculoskeletal disorders

Musculoskeletal disorders represent the most common occupational health problem among beedi workers in India, exceeding the burden of respiratory and dermatological conditions with pooled prevalence estimates reaching 76% in informal sector occupations, largely due to prolonged static sitting and repetitive hand movements (7, 8, 15, 17).

- *Low back pain*: This is the most commonly reported condition, affecting over 50% of workers in regions such as Telangana and Karnataka. It is largely associated with prolonged years of exposure and the lack of ergonomic support during extended 8–10-h working periods. Similar occupations involving sustained, hunched sitting postures have reported prevalence rates as high as 75–80% (7, 14, 18).
- *Neck and shoulder pain*: Neck pain has been reported in 45–63% of workers, while shoulder pain ranges from 41.7 to 75%. These conditions are mainly linked to sustained forward bending of the spine and repetitive reaching movements during work (14, 19).
- *Wrist and finger pain*: Pain in the wrist and hands is highly prevalent, ranging from 70.3 to 83.3%, while finger pain affects approximately 31.7–55% of workers. These symptoms arise from repetitive rolling movements, which can lead to cumulative trauma disorders such as carpal tunnel syndrome. The absence of protective measures further aggravates these conditions (7, 14, 19, 20).

Collectively, these findings highlight the substantial physical burden associated with this labour-intensive occupation, with nearly 90% of workers reporting fatigue and localized body pain (2). Emerging clinical evidence also suggests that such repetitive strain may progress to chronic inflammatory conditions and long-term joint damage if left unaddressed (1, 7). Despite this, awareness of these occupational risks remains low among workers, often delaying early diagnosis and limiting the adoption of basic preventive measures (2).

## 5 Ergonomic and occupational risk factors

In India’s largely decentralized, home-based beedi industry where more than 99% of production takes place within households women constitute the majority of the workforce and often face a dual burden of income generation and domestic responsibilities. Typically, they engage in 8–10 h of piece-rate work, producing between 200 and 1,000 beedis per day, often in the absence of adequate occupational safety measures. These structural conditions contribute to a markedly high prevalence of musculoskeletal disorders, with some systematic reviews in similar informal sectors reporting rates exceeding 76% (2, 6, 8).

The nature of the work requires prolonged sitting in unsupported postures, placing continuous strain on the spine. At the same time, repetitive and precise hand movements involved in rolling beedis exert considerable biomechanical stress on the wrists, fingers, shoulders, and neck. These risks are further intensified by poorly designed, makeshift work environments characterized by inadequate lighting, poor ventilation, and constant exposure to tobacco dust, often without the use of protective equipment. Additionally, low literacy levels, limited awareness of occupational hazards, and early entry into the workforce contribute to cumulative physical strain over time (2, 6, 7, 21–23).

These interrelated factors contribute to a high burden of chronic musculoskeletal disorders (MSDs), which emerge as the most common health problem among beedi workers, with reported prevalence ranging from 50 to 87%, exceeding that of respiratory conditions. Low back pain affects more than half of the workers, with some studies reporting rates as high as 76.6%. Similarly, neck and shoulder pain have been observed in 63 to 80.85% of workers, while wrist and hand pain range from 70 to 83% (2, 7, 15, 16, 24–26). These conditions often result in significant functional limitations, affecting daily activities in nearly 62% of workers and specifically impairing back-related functions in about 36%. The resulting loss of manual dexterity can reduce productivity and income, further aggravating their socioeconomic vulnerability. In addition, workers frequently experience multiple coexisting health issues, including fatigue and hypertension, along with an increased risk of long-term disability, particularly among those with more than a decade of occupational exposure. The piece-rate payment system further reinforces this cycle, as economic necessity often compels workers to prioritize output over health. Consequently, the gradual and often unnoticed physical toll remains unaddressed, perpetuating a cycle of exploitation and progressive functional decline in the absence of timely interventions (2, 27).

## 6 Preventive strategies and occupational health interventions

Addressing musculoskeletal disorders among female beedi rollers in this informal, home-based sector requires the implementation of low-cost and culturally appropriate interventions. These strategies should focus on practical ergonomic improvements, targeted health education, and simple exercise-based approaches, along with stronger policy support and community-level initiatives. Such measures need to be tailored to the realities of small-scale, home-based work environments, which are often characterized by poor lighting, inadequate ventilation, and limited access to protective equipment (2, 6, 28, 29).

### 6.1 Ergonomic modifications

The provision of simple ergonomic modifications, such as appropriate worktables and height-adjustable seating, can play a key role in reducing awkward postures commonly observed among beedi workers, including prolonged floor sitting and forward bending of the spine. By encouraging more neutral body positions, these interventions help minimize biomechanical stress on the back, neck, wrists, and fingers (29, 30). Evidence from similar labour-intensive informal sectors suggests that redesigning workstations to include adjustable heights, basic ergonomic supports for rolling activities, and improved seating can effectively reduce discomfort and lower the risk of musculoskeletal disorders, even with minimal financial investment (29, 30). Such low-cost adaptations are particularly relevant in home-based settings, where improvised work arrangements often contribute to repetitive strain and long-term physical burden (2).

### 6.2 Peer-led health education

Peer-led educational interventions can play an important role in improving awareness among beedi workers, particularly in low-literacy settings. These programs can focus on the use of basic protective measures, such as masks and gloves, along with simple hygienic practices like changing clothes after work. In addition, educating workers about their legal rights can help them better understand and address occupational risks, including exposure to tobacco dust and poor working posture (2, 3, 13, 31). Evidence from other high-risk informal sectors, such as tanneries, suggests that peer-based approaches are effective in promoting awareness and encouraging positive behavioural change through worker-to-worker communication (13, 32). Such strategies are especially useful in addressing the often “invisible” nature of occupational hazards and the low tendency among workers to seek medical consultation. Furthermore, incorporating basic knowledge of occupational health regulations can support self-advocacy and help reduce the lack of awareness regarding gradual health deterioration (2, 31).

### 6.3 Regular stretching and strengthening exercises

Incorporating simple daily stretching and strengthening exercises targeting the neck, upper back, shoulders, wrists, and lower back can help alleviate the cluster of musculoskeletal symptoms reported among 50–72% of workers (33, 34). Evidence from quasi-experimental studies conducted among workers engaged in similar repetitive tasks has demonstrated significant reductions in musculoskeletal disorder scores, such as those assessed using the Nordic Body Map, following such interventions (33, 34).

These exercise-based approaches, which do not require specialized equipment, can be easily integrated into routine work schedules and have been shown to improve posture and overall functional capacity. Promoting these practices through peer groups may further enhance adherence, particularly in settings where workers spend long hours typically 8–10 h per day in fixed postures that contribute to fatigue, discomfort, and dizziness (2).

### 6.4 Policy frameworks and occupational health walkthroughs

Strengthening policy frameworks is essential to address the occupational health risks faced by workers in the informal beedi sector. This includes enforcing basic safety measures, conducting regular occupational health assessments, and promoting alternative livelihood options in line with WHO recommendations (6, 28, 35). Periodic workplace assessments, as demonstrated in integrated Occupational Safety and Health (OSH) programs within the tobacco sector, can help identify key hazards such as poor ventilation and provide practical, context-specific solutions (28, 35).

In addition, supporting workers in transitioning to alternative sources of income—such as tailoring or small-scale farming, which have been preferred by a significant proportion of workers can reduce their dependence on beedi rolling (28, 35). Aligning these efforts with national policies, including India’s Occupational Safety Code, and global frameworks such as the WHO Framework Convention on Tobacco Control (FCTC) Article 17, can further strengthen monitoring mechanisms and facilitate the integration of rehabilitation and support services (6).

### 6.5 Community initiatives

Community-based initiatives can play a vital role in improving awareness and advocacy among women engaged in beedi rolling, who often shoulder both occupational and domestic responsibilities. Participatory approaches such as photovoice, which enable workers to document and reflect on workplace hazards, can strengthen collective understanding and encourage community-level action (22).

When combined with strengthened welfare schemes and skill-development opportunities, these strategies can help address the persistent issue of underreporting and promote more sustainable behavioural and occupational shifts. In the long term, such efforts may contribute to reducing the risk of disability associated with early entry into this occupation and prolonged exposure to adverse working conditions (2, 28).

## 7 Discussion

Musculoskeletal disorders among beedi workers are largely driven by poor ergonomic conditions and the precarious nature of informal labour. As a result, prevalence rates are alarmingly high, often exceeding 70% in key body regions such as the wrists, fingers, back, and neck (14, 23). These conditions arise from repetitive pinching and rolling movements, prolonged static postures typically 8–10 h of floor sitting with a forward-flexed spine and the absence of basic ergonomic support (15, 21, 40).

These risks are further intensified by the “invisible” nature of the industry. Since beedi rolling is predominantly home-based, it often falls outside the scope of formal workplace regulations related to workstation design, lighting, and occupational safety. Consequently, ergonomic issues are frequently perceived as individual problems rather than systemic concerns (6, 36). In addition, low levels of literacy and awareness mean that many workers attribute persistent pain to ageing, delaying timely recognition and intervention until the damage becomes more severe. Healthcare providers may also overlook the occupational origin of these symptoms, contributing to the underreporting of work-related musculoskeletal disorders (6, 43).

The burden of musculoskeletal disorders in this sector is comparable to, and in some cases exceeds, that reported in other Indian cottage industries. For instance, handloom weavers commonly experience back pain due to awkward working postures, while papad makers report chronic musculoskeletal issues linked to repetitive, fine motor tasks (14, 19).

Ultimately, it imposes profound public health and economic tolls on India through 4.9% annual household income losses from absenteeism and treatments (37), a gendered “double burden” on women juggling lost wages and domestic duties (24), and glaring healthcare gaps where only 17% access public clinics amid reliance on untrained providers demanding urgent targeted interventions for these invisible workers (36, 37).

The evidence highlights the devastating toll of musculoskeletal disorders on female beedi rollers, driven by ergonomic deficits and informal sector vulnerabilities, necessitating urgent, multifaceted interventions including low-cost workstation adaptations, ergonomic education, policy informal workers, strengthened primary healthcare support, and transitions to safer livelihoods to avert long-term disability and economic hardship (38, 39, 41, 42).

## 8 Future directions

Although many studies have reported a high prevalence of musculoskeletal disorders among female beedi rollers, much of the existing research is descriptive and cross-sectional, which limits a deeper understanding of the long-term health consequences of this occupation. Future research should therefore focus on longitudinal studies that can better explore how prolonged beedi rolling, early entry into the occupation, and continuous repetitive work contribute to chronic musculoskeletal problems over time. While several studies have identified ergonomic risks associated with beedi rolling, there is still a need to evaluate simple and affordable ergonomic interventions that can be implemented in home-based work settings. The largely home-based and informal nature of beedi rolling also leads to underreporting of occupational health issues, as workers often remain unaware of the occupational causes of their symptoms and may attribute them to aging. In addition, healthcare providers may overlook the occupational links due to limited awareness. Future research should therefore aim to generate stronger evidence that not only highlights the health burden but also supports the development of practical interventions and informs policies that protect the health and well-being of beedi workers.

## 9 Conclusion

This review draws attention to the extensive burden of musculoskeletal disorders among female beedi rollers, particularly involving the wrists, back, neck, and upper limbs. These conditions are largely driven by repetitive manual tasks, prolonged floor-sitting, and the absence of basic ergonomic support. The home-based nature of the industry often limits regulatory oversight, while low levels of education and the tendency to attribute symptoms to ageing further delay recognition and care. Together, these factors contribute to significant public health and economic consequences, including reduced earnings, the added strain of dual domestic and occupational responsibilities, and limited access to healthcare services. Addressing these challenges requires a comprehensive and context-sensitive approach. Occupational health strategies should prioritize low-cost ergonomic interventions, improved workstation design, and the incorporation of simple stretching exercises and task variation within daily routines. Integrating these measures with primary healthcare services can facilitate early identification and management of musculoskeletal conditions. At the policy level, stronger enforcement of occupational safety standards is needed, aligned with frameworks such as India’s Occupational Safety Code and the WHO Framework Convention on Tobacco Control (FCTC) Article 17, to improve monitoring and support alternative livelihood options. In addition, community-based initiatives including participatory approaches such as photovoice, peer networks, and local training programs can enhance awareness, encourage safer work practices, and promote timely health-seeking behaviour. Overall, a coordinated strategy that combines ergonomic improvements, policy support, and community engagement is essential to reduce the risk of long-term disability and improve the quality of life of women engaged in beedi rolling.
